# 2017 Dutch Report Card^+^: Results From the First Physical Activity Report Card Plus for Dutch Youth With a Chronic Disease or Disability

**DOI:** 10.3389/fped.2018.00122

**Published:** 2018-04-30

**Authors:** Marcella Burghard, Nynke B. de Jong, Selina Vlieger, Tim Takken

**Affiliations:** Shared Utrecht Pediatric Exercise Research Lab, Child Development & Exercise Center, Wilhelmina Children's Hospital, University Medical Centre Utrecht, Utrecht, Netherlands

**Keywords:** children, youth, chronic disease, disability, health, exercise

## Abstract

**Background:** The Dutch Active Healthy Kids (AHK) Report Card^+^ (RC^+^) consolidates and translates research and assesses how the Netherlands is being responsible in providing physical activity (PA) opportunities for youth (< 18 years) with a chronic disease or disability. The aim of this article is to summarize the results of the Dutch RC^+^.

**Methods:** Nine indicators were graded using the AHK Global Alliance RC development process, which includes a synthesis of best available research, surveillance, policy and practice findings, and expert consensus. Two additional indicators were included: weight status and sleep.

**Results:** Grades assigned were: Overall Physical Activity, *D*; Organized Sports Participation, *B–*; Active Play, *C–*; Active Transportation, *A–*; Sedentary Behavior, *C*; Sleep *C*; For Weight Status, Family and Peers, School, Community and Built Environment, Government Strategies, and Investments all *INC*.

**Conclusions:** The youth with disabilities spend a large part of the day sedentary, since only 26% of them met the PA norm for healthy physical activity. Potential avenues to improve overall physical activity are changing behaviors regarding sitting, screen time, and active play. The Netherlands is on track regarding PA opportunities for youth with disabilities, however they are currently not able to participate unlimited in sports and exercise.

## Introduction

According to the World Health Organization (WHO) physical inactivity is the fourth leading risk factor for mortality. Regular physical activity (PA) reduces the risk of many diseases including cardiovascular disease, diabetes, breast and colon cancer, and depression (1). Noting that the more physically active the child the greater the health benefit, specific research showed that PA has positive effects on musculoskeletal health, cardiovascular health, and mental health (2). It has been indicated as well that the earlier in life one starts engaging in sports and exercise, the longer one benefits from it (3). Therefore, PA is important. However, according to the Global Matrix 2.0, in which Report Cards from 38 countries, including the first Dutch Physical Activity Report Card, were compared regarding PA behavior, norms were often not met by typically developing youth (4). The Report Card is an annual update or “state of the nation” that assesses how a country is doing as a nation at promoting and facilitating PA opportunities for children and youth and grades outcomes using an academic letter grade approach (i.e., A, B, C, D, F). Data to grade the outcomes are drawn from several sources, including the research literature, governmental agencies, and non-governmental organizations^1^ (5).

Next to typically developing children, also many children with disabilities are not physically active (6). Even though it might be especially important for this group of children to engage in sports and exercise, because of the positive health effects in the physical, mental, and social domain (4, 7–10). Because of multiple barriers, this group should perhaps be more stimulated and encouraged to engage in an active lifestyle in a broad sense: from PA during sports and play activities and reducing sedentary behavior, to behavior related to sleep, and weight/nutrition (11).

Over the past few years, changes have occurred to facilitate the sports and exercise behaviors of people with disabilities in the Netherlands. Many organizations, foundations, and governmental bodies developed or funded projects that focus on improving PA and sports participation among people with disabilities. However, it is not yet clear what the overall effects of these projects were and where the gaps are. Do people with disabilities feel less restricted in the opportunities they have to participate in sports?

In the Netherlands, there was no overview yet of the actual status of PA behavior, sleeping behavior and weight status for youth with disabilities. Regarding the proven and potential positive effects of exercise for a good health, it was considered useful to fulfill this gap, by Active Healthy Kids the Netherlands, which consists of a group of researchers in the field of PA in children and youth, with a mission to inspire the nation to engage all children and youth in PA by providing expertise and direction to policy makers and public on how to increase, and effectively allocate resources and attention toward PA for Dutch children and youth. This is also the first Report Card in the world that was specifically developed for this group of children.

**Figure 1:**
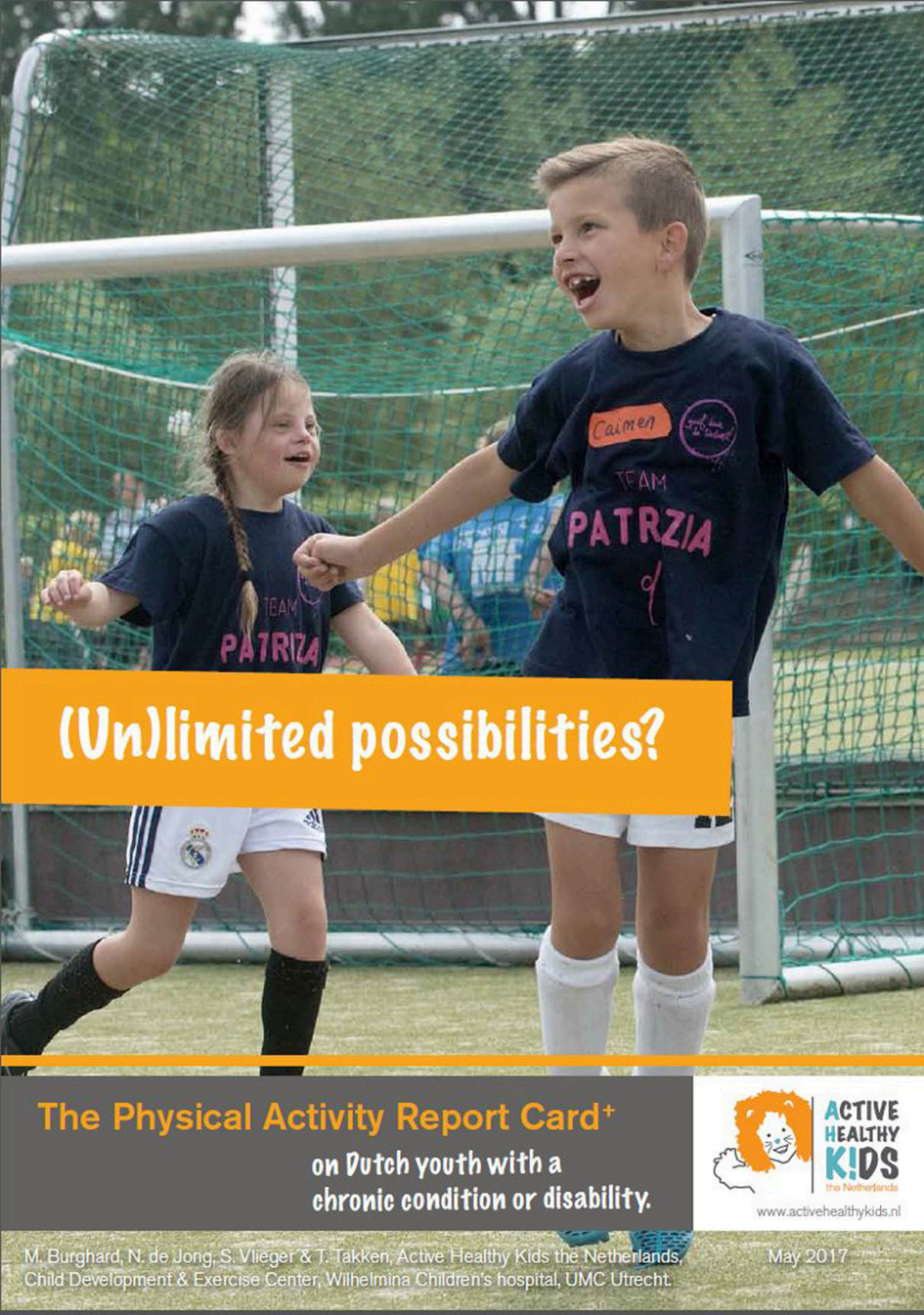
Front cover of the 2017 Dutch Physical Activity Report Card^+^.

With this Report Card^+^, we want to gain more insight in the PA levels and patterns of the Dutch youth with a chronic disease or disability and answer the question “*how (un)limited are the possibilities for the Dutch youth with disabilities to be physical active?”* (Figure 1).

In line with this, another aim was to compare the results of the Report Card for Dutch typically developing youth with the Report Card^+^ for Dutch youth with disabilities.

## Methods

**Figure 2:**
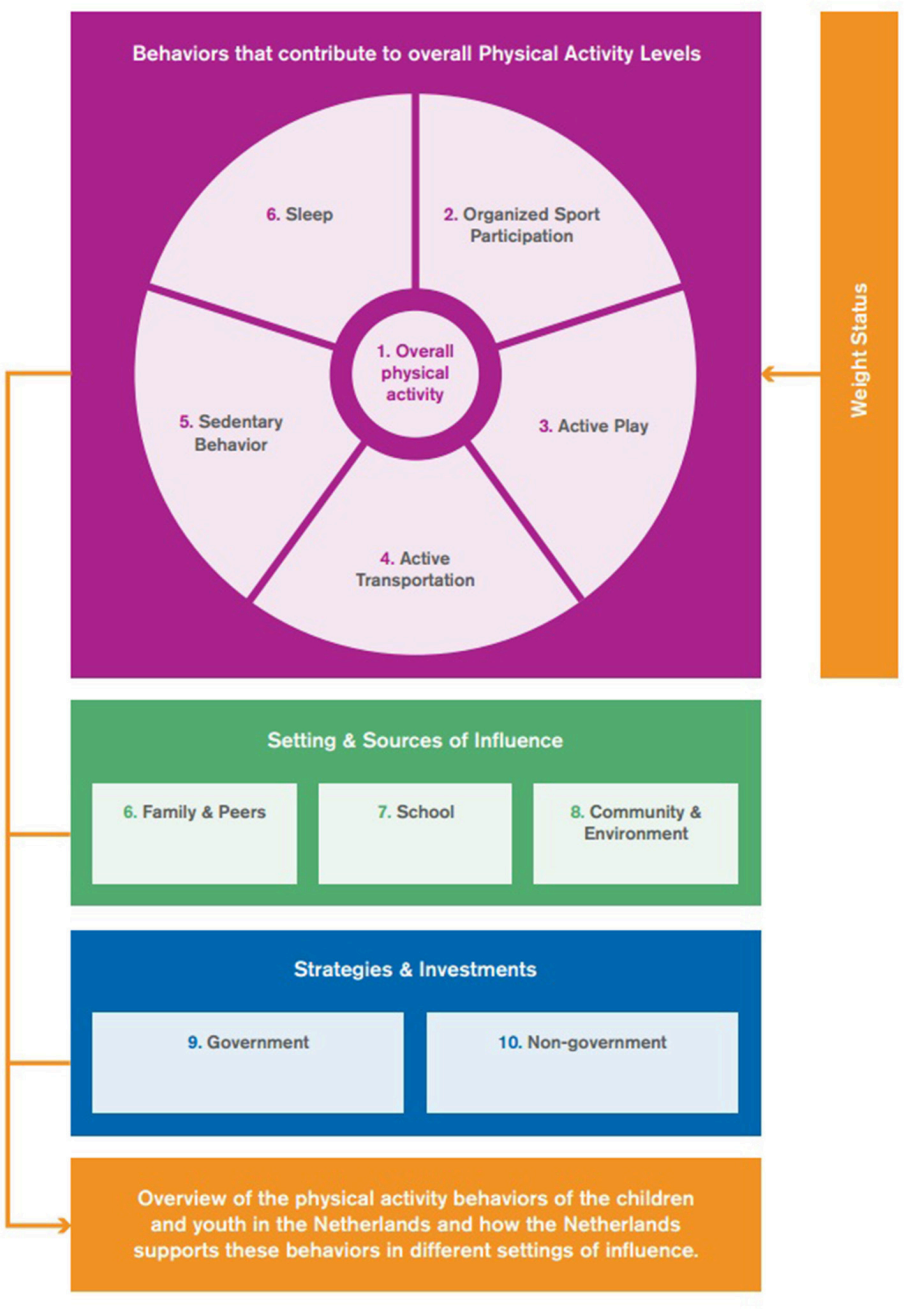
Overview categories and related indicators.

For the developmental process, guidelines of the Active Healthy Kids Canada framework were followed (5). Eleven indicators were graded in this Report Card. Nine of the indicators were part of this standard international framework. It was decided to add sleep behavior and weight status as additional indicators. The indicators were divided over three categories, except weight status, which did not fit in any of the categories (Figure 2). The grades were based on the percentage that met the single or multiple benchmarks.

The principal investigator and project manager formed a research work group (RWG) together with seven researchers of the University Medical Centre Utrecht, Utrecht University, Utrecht University of Applied Sciences and Center of Excellence in Rehabilitation Medicine Utrecht.

An expert group was formed with the involvement of National Institute for Public Health and Environment (RIVM), Mulier Institute, Dutch Olympic Committee^*^Dutch Sports Federation (NOC^*^NSF), Windesheim University of Applied Sciences, Knowledge Centre for Sports Netherlands (KCS), Hanze University of Applied Sciences Groningen, Amsterdam University of Applied Sciences, Institute for Health and Care Research, Netherlands Institute for health service research (NIVEL), and an advisory role for the Primary Education Board [PO-Raad].

Both the RWG and the expert group were responsible for the interpretation and evaluation of the data sources and evidence and had to decide about definitions and benchmarks of the indicators for the grading and were responsible for the final grading.

For the evaluation of the indicators, data of the period 2011 up to 2015 have been included. When available, we used data gathered by Statistics Netherlands (CBS) and the RIVM as the primary source. These organizations annually collect data about several lifestyle themes, the Lifestyle Monitor, part of which is the National Health Survey (NHS) (12). Most of the grading was based on this survey. The Lifestyle Monitor divides youth in two age groups: 4–11 years and 12–17 years. As a consequence of this, both age groups were assessed for each indicator. Unfortunately, the sample sizes for the years 2011 up to 2014 were too small for a subgroup analysis. For 2015, 142 children were included for the age range 4–11 years and 232 children were included for the age range 12–17 years. For the youngest age category (4–11 years), the answers were parent reported. For the older age group (12–17 years) the NHS was a self-report questionnaire. If the required data to grade an indicator could not be provided by the primary sources, other governmental and non-governmental sources were used.

Children with disabilities in the Netherlands could attend regular education or special education at special schools. The situation of scholars attending special education was described when reports were available (13). The grades of the indicators were based on the data about youth with disabilities in general from the NHS.

As this Report Card^+^ was developed following a standard framework that was also used for the first Dutch Physical Activity Report Card for typically developing children, the results of both Report Cards could be compared.

### Benchmarks

The first six indicators are Overall Physical Activity, which is also the first category, and the behaviors that contribute to that: Organized Sports Participation, Active Play, Active Transportation, Sedentary Behavior, and Sleep. For all of these indicators the grading was based on data from the NHS for children with disabilities in general and, when available, data from the Mulier Institute were used to describe the situation of children attending special education (13).

For the first indicator, Overall Physical Activity, the grading was based on the percentage of children who met the Dutch Physical Activity Guideline [NNGB: Dutch Guidelines Healthy Physical Activity; to be at least moderate active (at least 5 MET) for at least 60 min every day]. For this indicator data on children attending special education were available.

The RWG and expert group reached consensus to use data regarding engaging sports on a weekly basis, thus the grading of Organized Sports Participation was based on the percentage of children and youth who participated in organized sports and/or PA programs weekly.

For children attending special education, it was known how many children were a member of a sports club and how many played non-school based sports at least once a week. Regarding Active Play, the grading was determined by the percentage of children who played outside for at least 60 min after school, for 7 days a week. The NHS does not include questions about active play behavior in 12–17 year old youth, therefore the grade was based only on 4–11 year old children. For the scholars attending special education the percentages of children who played outside 5–7 times a week were reported.

Active Transportation was assessed by the percentage of children who use active transportation (walking and cycling) to get to and from places (school and/or work) for at least 3 days a week. Of children attending special education, only the amount of children who used active transport was known and not the weekly frequencies.

For Sedentary Behavior only the amount of time spent in front of a screen (screen time) was surveyed, so the grading was based only on this criteria even though this does not cover all sedentary time. The number of children who watch television or sit in front of the computer less than 2 h a day outside school hours determined the grade for this indicator. No numbers where available on sedentary time of scholars attending special education. The indicator Sleep was assessed by the amount of children meeting the sleep duration recommendation for their age group. The sleep duration recommendations used are described in the study of Hirshkowitz et al. (14). These recommendations are for healthy individuals with normal sleep. The appropriate sleep duration for school-aged children is considered between 9–11 h each night and for adolescents this is 8–10 h (14).

For the additional indicator Weight Status, the grade was based on the percentage of children with a normal body weight (BMI between 18.5 and 25 kg/m^2^ was classified as normal weight) (15). Data for this indicator were taken from the NHS for the children with disabilities in general. Data of scholars attending special education were used from reports from the Mulier Institute (13).

The next category, Settings and Sources of Influence, consists of the indicators Family and Peers, School and Community and Environment. No data of the NHS regarding these indicators were present. Thus, no general information was present. Other sources were used for assessment of the indicators in this category.

The criteria of Family were: “percentage of parents who facilitate PA and sports opportunities for their children (e.g., volunteering, coaching, driving, paying for membership fees, and equipment),” “percentage of parents who meet the PA guidelines for adults” and “percentage of parents who are physically active with their kids.” For Peers the criteria were: “percentage of children and youth with disabilities with friends and peers who encourage and support them to be physically active” and “percentage of children and youth who encourage and support their friends and peers to be physically active.” However, as there was no consistent data for children with disabilities in general nor for children attending special education (not all clusters^2^), the RWG and experts decided that this indicator could not be graded. The available numbers of some of the clusters in special education from the Mulier Institute and other sources were used to get some insight in this indicator.

For School the following criteria were set: “the percentage of schools with an active school policy (e.g., offering sports- and exercise activities next to physical education (PE) or activities during recess, collaboration with communities and/or sports clubs, presence of annual planning),” “percentage of schools with a PE specialist,” “the percentage of schools were the students have at least 90 min of PE per week,” and lastly “the percentage of students who have at least 45 min of outside play time during school for 5 days per week.” Again, however, it was decided to grade this indicator as Incomplete. Data was present about regular education and special education. However, as a consequence of the regulation “Appropriate Education” [Wet Passend Onderwijs]^3^, some children with disabilities attended regular schools and participate in regular PE. The specific situation for these children was unknown.

The last indicator of this category, Community and Environment, also had several criteria: “the percentage of children and parents who perceive their community/municipality is doing a good job at promoting PA (e.g., variety, location, cost quality),” “the percentage of communities/municipalities that report they have policies promoting PA,” “the percentage of communities/municipalities that report they have infrastructure (e.g. sidewalks, trails, paths, bike lanes) specifically geared toward promoting PA,” “the percentage of children or parents who report having facilities, programs, parks, and playgrounds available to them in their community,” “the percentage of children or parents who report living in a safe neighborhood where they can be physically active,” “the percentage of children who report having well-maintained facilities, parks and playgrounds in their community that are safe to use” and finally, “the percentage of children and parents who report that in organizations like sports clubs, they (their child) are socially accepted and that social accessibility is present.” Also for this indicator it was decided to mark it as an Incomplete. In the Report Card for typically developing children, data of the Leisure time Omnibus [Vrijetijdsomnibus] of the CBS and the Netherlands Institute for Social Research (SCP) was used to grade this indicator. Unfortunately, the sample size of children with disabilities was too low for both 2012 and 2014 to use the results.

The last category, Strategies and Investments was divided in the indicators Government and Non-Government. The criteria that were set were: “evidence of leadership and commitment in providing PA opportunities for all children and youth,” “allocation of funds and resources for the implementation of PA promotion strategies and initiatives for all children and youth” and “demonstrated progress through the key stages of public policy making (i.e., policy agenda, policy formation, policy implementation, policy evaluation, and decisions about the future).” No clear numbers were available to state that policy is efficient or how much financing is acceptable. Therefore, the decision was made to grade this indicator with an Incomplete. Multiple governmental documents were studied and reports of the Mulier Institute on different policies and programs were evaluated. For Non-Government, annual reports and websites of several national and regional foundations and organizations were considered.

The RWG and experts evaluated the evidence for each of the indicators and discussed the proposed grading. The grades were based on the percentages of youth meeting the defined benchmark. Some indicators are stand-alone, while others are comprised of several components. A was 81 to 100%, B was 61 to 80%, C was 41 to 60%, D was 21 to 40%, F was 0 to 20%. INC was incomplete data or not enough available evidence to assign a grade to the indicator or absence of clear well-established criteria. This grading system is in accordance to the Canadian Report Card framework (5).

When the data about scholars attending special education showed that the situation for that particular indicator was considerably better or worse for these children, the grade was given a plus or minus respectively.

## Results

The 2017 Dutch Report Card^+^ is the first ever assessment of PA behaviors, settings, and sources of influence and government strategies and investments for children with a chronic disease or disability. The grades are summarized in Table 1.

**Table 1:** Overview of indicators and corresponding grades.

**Indicator**	**Grades**
Overall physical activity	D
Organized sports participation	B–
Active play	C–
Active transportation	A–
Sedentary behavior	C
Sleep	C
Weight status	INC
Family and peers	INC
School	INC
Community and the built environment	INC
Government strategies and investments	INC

### Overall physical activity levels: D

The grade for Overall Physical Activity levels was a *D*. In 2015, 26% of both children and youth (4–17 year olds) met the Dutch PA guideline of Healthy Physical Activity (NNGB). Scholars of cluster II schools were the most physically active compared to the other clusters. Of the scholars attending cluster II schools 35% exercised 8 or more hours per week (excluding sports) (13). For the cluster I and III scholars this was 21% and in cluster IV 27% (13).

### Organized sports participation: B–

Of the 4–11 year olds 69% and of the 12–17 year olds 73% was considered a weekly athlete (12) Among scholars attending special schools, the sports participation was lower. Cluster IV scholars had the highest sports participation, namely 45 vs. 25, 37, 26% for cluster I, II, III, respectively (13).

### Active play: C^–^

Of the 4–11 year old children with disabilities 53% played outside for at least 60 min after school, on all days of the week (12). Scholars of cluster II schools, most often played 5–7 times per week outside (45%), compared to cluster I (31%), III (30%), and IV (33%) scholars. The average amount of minutes of active playtime outside school hours was 529 min per week for the 4–11 year old children with disabilities (13).

### Active transportation: A–

Of the children in the age of 4–11 years 39% cycled 3 or more days to or from school or work and this was 38% for walking 3 or more days per week.

Of the 12–17 year olds 71.8% cycled 3 or more days to or from school or work and this was 15.8% for walking 3 or more days per week (12).

Only 4% of the children in cluster I schools used active transportation to get to their school (13). This was 18% in cluster II, 13% in cluster III, and 30% in cluster IV schools (7, 13).

### Sedentary behavior: C

Of the 4–11 year old children 45.5% sat in front of the computer or watched TV, less than 2 h a day (average day of the week), outside school. This was only 23.2% for 12–17 year old children (12). No data concerning sedentary behavior was available for scholars in special schools.

The 4–11 year olds sat/lay on average 7.9 h per day on a school day, compared to 11.1 h for the 12–17 year olds. On a day off from school, the younger age group sat/lay on average 6.5 h, compared to 9.2 h in the older age group (12).

### Sleep: C

Of the 4–11 year old children with disabilities 26% met the sleep recommendations. This was 63% in the 12–17 year old age group (12). No data was present about sleep behavior of scholars attending special schools.

### Weight status: INC

The sample size of the NHS was unfortunately too small, to grade this indicator. These data showed however, that the mean BMI of the 4–11 year olds was 16.5 and 20.8 kg/m^2^ in the 12–17 year old age group (12).

When evaluating the scholars who attended special schools (all clusters together), 68% of the children had a normal weight, 11% was underweight, 17% was overweight, and 4% obese. When comparing the different clusters, the highest percentage of overweight and obese children (combined) was found in cluster III schools (25%) (13).

### Family and peers: INC

No data of the NHS regarding “Family & Peers” were present. Thus, no general information was present to grade this indicator. Data was only available on parents of children in cluster III or IV schools. No information about the parental behavior in the other two clusters was present, consequently an Incomplete was graded. Of the parents of cluster IV scholars 59% considered it important that their child engages in sports or exercise frequently. Of the parents 72% encouraged their child to play sports or exercise frequently (8). A smaller study showed that parents of whom the child joins a sports club, stimulate their children significantly more (*p* = 0.05) to sports and exercise, than parents whose child is not a sports club member (9).

### School: INC

Data was present about regular education and special education. However, as a consequence of the regulation “Appropriate Education” [Wet Passend Onderwijs]^3^, some children with disabilities attend regular schools and participate in regular PE. The specific situation for these children was unknown and consequently an Incomplete was graded. Key findings about the situation in special schools will be given.

Concerning active school policies, 71% of the special schools offered their students other sports and exercise activities, next to PE (10). All cluster I and II schools, had a PE specialist, and 84.2 and 94% of the cluster III and IV schools had a PE specialist respectively. All cluster schools offered twice a week PE (7, 8, 13) The number of average minutes PE per week varied between 63 min per week in cluster III to 103 min in cluster IV (7, 8).

Regarding playtime during school recess, 50% of the 4–11 year old students played at least 45 min outside during school time for 5 days per week and the average active play time at school was 284 min per week for this age group (12).

### Community and the built environment: INC

As mentioned in the methods, the sample size of children with disabilities in the Leisure time Omnibus [Vrijetijdsomnibus] of the CBS and SCP was unfortunately too low to grade this indicator. A smaller study showed that 12% of the parents of children with disabilities reported that play sets/grounds are not nearby enough. Only 2% of these parents reported that the play sets/equipment are not safe and/or badly maintained and only 1% considered them not safe (for younger children). Only 9% of these parents reported that it was not safe for their children to play in the neighborhood, due to traffic safety (16).

### Government strategies and investments: INC

This indicator about the current policy of the government could not be judged. There have been several initiatives that have to resulted in a more physically active youth. Unfortunately, no clear criteria and monitors were present to evaluate the effectiveness of these initiatives and policies.

With regard to foundations, we saw that proportionally more foundations were founded to help or facilitate children with disabilities in their possibilities to play sports or exercise compared to foundations for typically developing children.

## Discussion

The primary aim of this Report Card^+^ was to provide an overview of the methods and results of the first Dutch Report Card^+^ for youth with disabilities. The results showed that about a quarter of the Dutch youth with disabilities met the PA norm.

**Figure 3:**
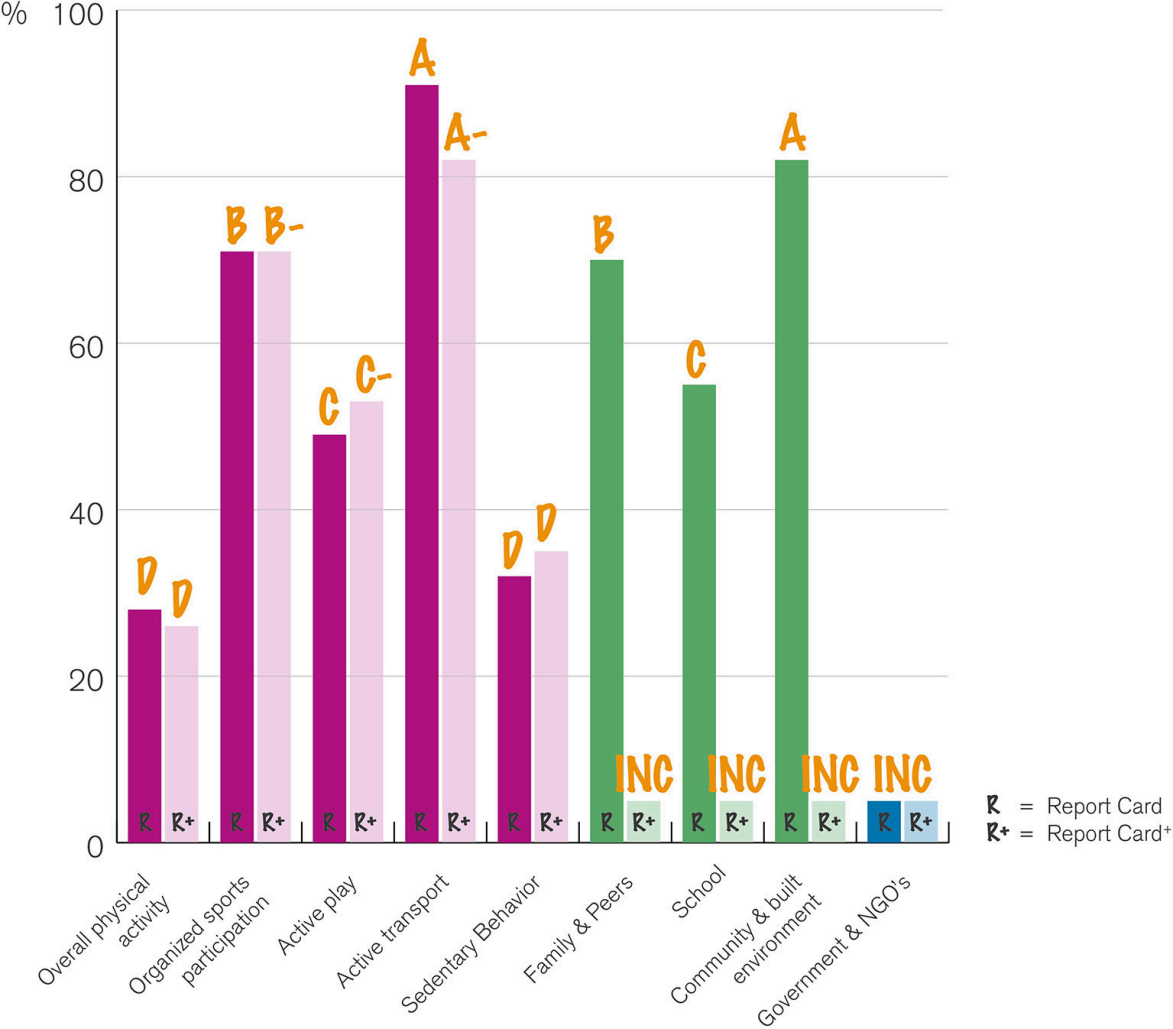
Comparison of the results of the Report Card and the Report Card^+^.

In 2016 the results of the first Dutch Physical Activity Report Card were published (17). These results were compared with the Report Card^+^ results (Figure 3). A notable finding was that the percentages of children that met the Dutch Physical Activity Guidelines was the same for children with and without disabilities (26%). Assessing the different indicators that contribute to Overall Physical Activity (Organized Sports, Active Play, Active Transport, and Sedentary Behavior), it was clear that the youth with disabilities used active transport less often than their typically developing peers. Regarding youth attending special education, norms were less often met than in youth attending normal education. The differences between healthy children and children attending special education may be caused by the (social) accessibility and by the diversity of disorders/disabilities. Noteworthy, was that in the Report Card^+^, only six of the 11 indicators could be graded and five were graded an Incomplete, thus we stated that the national monitoring in youth with disabilities is unfortunately lacking. Therefore, it was difficult to make powerful statements about possible causes (17).

Other indicators for which improvement is warranted are sedentary behavior and active play. The Dutch youth with disabilities spent a large part of the day sitting or lying and/or behind a screen, especially during school times. Though, around half of the children with disabilities engaged in daily active play for at least 60 min, the other half did not. Thus, changing the behaviors regarding, sitting (at school), screen time, and active play, seems most likely to improve overall activity levels.

Fortunate, a large part of the youth with disabilities engaged in sports weekly and chose an active mode of transportation for their way to school. It is important that the conditions for these indicators will remain this high in the future. Solutions should be developed to make it possible for more scholars in special schools to travel to school (partly) using active transportation. Furthermore, sports clubs need to educate their staff and volunteers more properly so children and their parents experience less barriers to join a sports club.

The role of the parents and family is of high importance as well in this group of children. Even though no grade could be assigned to this indicator, results demonstrated that parents should be more informed about their large influence as a role model for all behaviors and that their home rules are of high relevance as well. Stimulating parents to engage in sports and exercise activities with their whole family should be more promoted. In addition, strategies that promote sports opportunities for children with disabilities, such as sports and play activities in the neighborhood and foundations who can help families with less financial back up, should be improved. Currently, too many children and parents are not familiar with these possibilities and sports opportunities.

As the indicator sedentary behavior showed, the youth with disabilities sat the most during school hours. Strategies to interrupt the long sitting duration should be developed and implemented, for example physically active academic lessons. As school is the place where all children can be reached, strategies, and financial resources are needed to enlarge the duration of PE lessons and to realize higher intensities during these lessons.

Further, collaborations between all sectors should be stimulated. Problems in the accommodation and offer of sports and other active activities will benefit from this. Furthermore, it is important to involve parents, PE specialists and teachers in realizing and improving the sports opportunities for children with disabilities. Both parents and teachers know the child and his/her possibilities and disabilities the best and can search together with the sports clubs for the most appropriate sports activity.

### Strengths and limitations

This is the first ever developed Physical Activity Report Card^+^ for children and youth with a chronic disease or disability. This Report Card provides a comprehensive overview about how the Netherlands is doing, regarding PA opportunities, overall PA levels and the role of sources of influence for children with disabilities.

Strength of this Report Card^+^ is the participation of many experts and organizations in this area, which made that many important data sources were identified and included. Unfortunately, not all indicators were integrated in national surveys yet (e.g., family and peers) and in the national surveys no clear demarcation was present for children with disabilities. No subcategories could be made and the size of the researched population is small. Furthermore, only the data of 2015 from the NHS could be used because the sample sizes in the years 2011-2014 were too small to analyze. With this in mind, one can question whether these results actually represented the current situation for people with disabilities and youth in particular. Making appropriate policies based on the results of this monitoring should therefore be questioned.

## Conclusion

Based on the results of this Physical Activity Report Card^+^, only 26% of the Dutch youth with a chronic disease or disability met the current national PA guidelines. The most important behaviors to change that will most likely result in improvement of overall PA levels seem to be sitting (at school), screen time, and active play. In the past few years, many initiatives, possibilities, and policies were developed and the Netherlands is on track, but currently, the Dutch youth with disabilities is not yet able to participate completely unlimited in sports and exercise.

## Additional information

The long form Report Card^+^, with more background information about the developmental process, methods, indicators, and recommendations, is available online: http://www.super-lab.nl/reportcarddownloads/.

## Author contributions

TT was the principal investigator and MB was the project manager according to the international Report Card framework. NdJ and SV supported TT and MB in their work in the Report Card developmental process (for example, literature search, analyzing the results, writing of final Report Card, and the manuscript).

### Conflict of interest statement

The authors declare that the research was conducted in the absence of any commercial or financial relationships that could be construed as a potential conflict of interest.

## Footnotes

^1^For a more detailed description about the Report Card, see Colley et al. (5)

^2^**Cluster I:** Schools for visual impaired children or children with multiple disabilities who are visually impaired or blind. **Cluster II:** Schools for deaf children and hearing impaired children, children with speech or language difficulties and children with communicative problems, as with some forms of autism. **Cluster III**: Schools for children with motor and/or mental disabilities, chronically ill children and children with epilepsy. **Cluster IV**: Schools for children with psychiatric disorders or severe behavioral problems and schools that are related to pedagogical institutes.

^3^In 2014, the regulation “Appropriate Education” [Wet Passend Onderwijs] was introduced, which aims that every student should attend a school that provides education suited to their talents and capabilities. Schools should adapt their teaching to the individual child's development and offer extra assistance. This applies to the school where the child is currently registered, another mainstream school or a school providing special education (16).

## Abbreviations

BMI: Body mass index
CBS: Statistics Netherlands
INC: Incomplete
KSC: Knowledge Centre for Sports Netherlands
MET: Metabolic equivalent
NHS: National Health Survey
NIVEL: Netherlands Institute for health service research
NNGB: Dutch Physical Activity Guideline
NOC^*^NSF: Dutch Olympic Committee^*^Dutch Sports Federation
PA: Physical activity
PE: Physical Education
RIVM: National Institute for Public Health and Environment
RWG: Research work group
SCP: the Netherlands Institute for Social Research
WHO: World Health Organization.
